# Particularities of Telework Applicable to the Health System in the Context of the COVID-19 Pandemic

**DOI:** 10.3390/ijerph191710501

**Published:** 2022-08-23

**Authors:** Alina Filip, Alin Stancu, Mihai Mehedințu, Adrian Streinu-Cercel, Alexandrina Maria Pauceanu

**Affiliations:** 1 Department of Marketing, Bucharest University of Economic Studies, 010374 Bucharest, Romania; 2 Faculty of Medicine, Carol Davila University of Medicine and Pharmacy, 050474 Bucharest, Romania; 3 Doctoral School and Research Center, Geneva Business School Spain, Carrer de Rivadeneyra, 4, 08002 Barcelona, Spain

**Keywords:** COVID-19, medical system, telework, qualitative marketing research, doctor-patient relationship

## Abstract

The paper aims to highlight how physician-patient relationships have evolved amid the COVID-19 pandemic by considering telework implementation into the healthcare sector. The article presents the peculiarities of using telework within the medical system given the recent epidemiological context, by pointing out the advantages and disadvantages of its adoption. To achieve this goal, a qualitative marketing research was conducted to identify physicians’ opinions and perceptions on telework. The main objectives were identifying the ways of using telework, the effects that telework has on the quality of the medical services and on patient relationships, as well as the strengths and weaknesses of telework for the medical field. The study revealed that while face-to-face consultations decreased as the outbreak continued, different methods of remote consultations emerged, which was both beneficial in interactions with chronic patients and detrimental, as medical staff became more and more overworked. For these reasons, our research shows that healthcare professionals consider a hybrid system much more adequate for patients with stable chronic conditions, as ongoing monitoring is done through this remote mechanism.

## 1. Introduction

From its beginning, the COVID-19 pandemic has led to many health, economic, and social consequences, getting to be considered a global health crisis [1]. Due to physical and social distancing measures implemented by governments, citizens were forced to make major and immediate lifestyle changes: how they interact with others, their buying patterns and consumption behavior, ways of socialization and entertainment, their relationship with their employers, and the forms of work practiced.

The use of information and communication technology (ICT) significantly increased during the COVID-19 pandemic, often being the only way to maintain social ties with community members and membership groups [2], as well as to ensure ongoing economic activities or, in some cases, even business survival [3].

The importance of ICT was recognized by more and more employers as a natural consequence of the restrictions adopted by governments which, in an attempt to limit physical interactions and decrease employees’ exposure to the risk of infection with the new coronavirus (SARS-CoV-2), required companies to adopt telework whenever possible [4].

Thus, many individuals were forced to quickly adapt to working from home and tackle the technological challenges for which they previously received little to no training. In addition to the pressure of working in a new climate, more or less equipped with the resources and technology needed for remote interactions, some people were also faced with challenges in managing new situations within their own families or households.

Consequently, if before the pandemic telework was just narrowly practiced (for example, in 2019, only about 5.4% of people employed in European Union countries were working from home [5]), by the end of 2020, studies showed that 39.6% worked from home [6]. Once the restrictions started being lifted, the percentage of people working from home decreased to 25% in 2021, and to 12% in the spring of 2022 [5], but hybrid work became commonplace in many businesses.

This very different situation raises fewer and fewer doubts about the permanent changes caused by the COVID-19 pandemic on employee-employer relationships, as well as on the need for telework in many economic sectors and the role of ICT in ensuring the sustainability of the desired productivity. Telework is currently a reality for many employees, and businesses started to implement changes to job policies, given the increased likelihood of an ongoing deployment of telework practices in the future, after overcoming the current health crisis.

The medical sector was not among the pioneers in adopting telework. In fact, prior to the emergence of SARS-CoV-2, there were rather isolated initiatives for the practice of telework in this field of activity, given the importance of face-to-face doctor-patient relationships in the processes of diagnosis, treatment, and monitoring of individuals. However, the COVID-19 pandemic has influenced working practices, including the medical sector, with telemedicine proving its effectiveness in controlling infection rates with the novel virus, ensuring patients’ access to specialized expertise, streamlining the treatment of patients with chronic diseases, as well as enabling work from home for doctors who were unable to travel to the health unit if self-isolating.

Compared to other areas, adopting telework in the healthcare sector raises several additional challenges, the most important being related to the difficulty of accurately identifying and adequately meeting patients’ needs in the context of lack of face-to-face interaction, as well as the implications of remote triage of patients, remote consultations, and remote patient monitoring.

The appearance of the first case of SARS-CoV-2 infection, reported on February 26, 2020, led the Romanian health system through a whole set of transformations, which significantly influenced the doctor-patient relationship. The decree of a state of emergency [6], which restricted both the movement of people and the activity of public hospitals in serving patients, and, later, the limitation of the number of hospitalizations and outpatient consultations [7], meant that the medical system needed to find a different way to interact with over 3 million chronically ill people in Romania. Such challenges have been reported in patients diagnosed with Hepatitis C or HIV/AIDS whose access to hospital care was hampered by converting departments of entire hospitals into COVID-19 -only units. 

In order to improve access to medical services in the context of epidemiological risk and spread of SARS-CoV-2, the Romanian Government [8] issued an emergency ordinance regulating telehealth in order to give the possibility to provide medical assistance through telemedicine by public and private health units. In addition, the list of medical services that can be provided via telemedicine was detailed by methodological norms [9]. Therefore, among the medical services that can be provided through telemedicine are teleconsultations for establishing the diagnosis, preventive health monitoring, interpreting results of medical investigations, establishing treatment, indicating methods of disease prevention in specialties such as infectious diseases, endocrinology, gastroenterology, ophthalmology, pediatrics, as well as health services related to medical care provided by psychologists.

The aim of the paper is to highlight the development of physician-patient relationship in the context of the COVID-19 pandemic by including the specific components of telework. The paper is divided into three sections, starting from the analysis of the literature in the field of telework and the development of research questions, continuing with the presentation of the qualitative research methodology conducted among doctors in a hospital specialized in infectious diseases and the results of the study. In the end, a series of conclusions and implications of the adoption of telework for the medical system, patients, and physicians are presented.

## 2. Literature Review

Telework is a relatively new economic concept, and this is probably why researchers have not reached a unanimous opinion on its significance [10]. The characteristics of the term are usually highlighted by comparison with classic working practices, traditionally carried out at business headquarters. Thus, the main particular features of telework are considered to be: distance from the conventional workplace [11], the use of ICT tools, such as tablets, laptops, computers, and smartphones, used specifically to work outside the employer’s office [12]; and flexibility in organizing an employee’s work schedule, in order to achieve a better work-life balance [13].

As can be seen, information technology is considered a necessary condition in the practice of telework, as it allows for reconfiguring and optimizing working tasks, communicating between colleagues or maintaining connections between team members, employee access to documents, and professional applications for carrying out current activities. All of these have, in many cases, a positive impact on labor productivity and reduced employer spending [14].

According to research in the field [15], teleworking brings several benefits to both employees and employers. The positive influence on the behavior and attitude of employees consists mainly in: increasing job satisfaction [16]; improving morale; increasing autonomy in carrying out professional activities, with an influence on increasing the flexibility of the working schedule and the well-being of employees [17]; diminishing absenteeism; avoiding distraction through the activities of others in the physical office and better focus on individual tasks [18]; reduction of labor disputes [19] (thanks to the lower number of interactions with colleagues and the performance of professional tasks in a familiar and relaxing climate); and the possibility of using technology to achieve a satisfactory mix between professional requirements and family needs [20].

Employers can also gain significant benefits by developing internal policies to regulate the way in which employees work remotely. Increasing productivity is one of the most significant economic benefits [21], along with decreasing the rate of employee turnover, and the need for smaller office space (and thus reducing the cost of renting office buildings).

On the flipside, recent research analyzing working from home in correlation with the notion of techno-stress [22] highlights the stress caused by continuous use of ICT and overworking, with no clear difference between work schedule and leisure time, with unfavorable consequences for private life. Thus, although the use of technology provides more flexibility in planning both work and family time, employees are at the same time under additional pressure to be constantly connected and receptive to possible demands that may arise throughout the day [23].

Other disadvantages of teleworking are developing feelings of alienation or isolation due to distance and lack of interactions with other people [24]; difficulty in accomplishing group tasks, which involve interdisciplinary consultations; and the need for frequent communications with team members and the extra time needed for this purpose [14].

The COVID-19 pandemic has highlighted the benefits of teleworking for both patient safety and front-line health care workers by preventing SARS-CoV-2 infections. Moreover, given that the medical system in many countries has faced major capacity constraints throughout the pandemic, telework can be the solution to cover unmet demand from health care consumers.

The sudden increase in hospitalization rates of COVID-19 patients led to a decrease in the availability of medical staff, in many healthcare units, in relation to chronic patients. In addition, worldwide, healthcare professionals have increasingly faced a phenomenon of physical overload and fatigue, which led governments and medical units to reinstate retired nurses and doctors. In this context, telework can be used to allow quarantined medical staff with mild or asymptomatic symptoms to provide support for overworked medical staff. In addition, specialized staff in areas with lower demand could be involved in providing virtual consultations and support to healthcare professionals in areas with higher demand [25].

Telework has the potential to streamline the doctor-patient relationship. For many patients, getting an accurate and quick diagnosis involves a complex process, with difficulties that may arise both in terms of the availability of the doctor and the time required to establish a physical consultation, and in terms of time spent in waiting rooms. Since the beginning of the pandemic, these barriers have been intensified by restrictions on mobility and quarantine, as well as by people’s fear of getting infected. Telework can be a way to decrease these issues and redefine patient interactions within safe environments. Of course, there are differences in the effectiveness of telework depending on the medical specialization and the complexity of patient’s needs. Some studies proved the main applicability of telework in the field of psychiatry and respiratory therapy [26].

## 3. Research Questions

The doctor-patient relationship can be mediated through multiple means of interaction. The concept of telemedicine, i.e., the use of technology to deliver remote healthcare [27], can be implemented in various medical specialties or clinical cases and is seen as having great potential in epidemic situations to improve epidemiological examinations, disease control and clinical care management [28]. The powerful role of telemedicine in the management of the COVID-19 pandemic has been presented in various studies [29,30,31], highlighting that in such situations, telemedicine is advantageous in terms of diagnosis, treatment, and patient follow-up, bringing substantial benefits to both physicians and patients.

Furthermore, in the case of the medical system, there are additional accepted concepts for remote working, such as remote consultation, which had a significant role in improving patients’ access to medical care during the COVID-19 pandemic, with a rapid upsurge in their use, conducted by physicians over the phone or through various video-calling applications [32]. The shift to remote consultations during the COVID-19 pandemic has been deemed a success, concentrating primarily on vulnerable patients, in order to limit the transmission of the virus [33]. In addition, through remote patient monitoring systems, the ability to remotely monitor the status of patients [34] and physiological data such as EKGs, EEGs, or blood oxygen monitoring were obtained from patients, physicians having access to updated data, in real-time, regarding the health status of their patients [35].

In this context, the purpose of this paper is to highlight the challenges that medical staff faced in the treatment of chronic patients due to the intensifying restrictions caused by the COVID-19 pandemic in Romania and how different ways of interaction with patients were employed, including telework, in order to satisfy care needs.

The research addresses the following research questions:

What was the impact of telework on the working schedule of doctors?

What methods were used by doctors in order to interact with their patients during the COVID-19 pandemic?

Which are the benefits and limitations of telework in medical services and in doctor-patient relationships?

The added value of our study consists in a better understanding of the potential impact of telework adoption in the healthcare system, identifying the advantages and disadvantages of this adoption and pointing out some recommendations could determine public health officials to revise the policies in this field. The findings of this study are also important for academics, medical staff, as well as for healthcare beneficiaries. 

## 4. Research Methodology

### 4.1. Study Design

The study aims to investigate physicians’ opinions on using alternative ways of patient interaction (e.g., telework) during consultations, considering the changes generated by the COVID-19 pandemic. The applied method is the qualitative marketing research. Semi-structured in-depth interviews were used to determine the transformations that took place in the doctor-patient relationship. The research is exploratory by nature, given the study of a recent phenomenon, but with major implications for the functioning of the health system and the provision of medical services needed by the population [36].

The hospital where the research was carried out is the largest infectious diseases hospital in Romania, being the first state health unit certified ISO 9001/2000 for the quality of specialized medical services. Before the outbreak of the COVID-19 pandemic, the hospital offered monitoring and treatment for patients with chronic diseases and had approximately 700 beds and included several clinical wards for adults and children, as well as intensive care wards, offering both continuous and one-day hospitalization. With the outbreak of the pandemic, it was converted into a first-line hospital for the treatment of COVID-19 patients, making it the largest COVID-19 hospital in Romania. All wards of the hospital became exclusively COVID-19 wards, except for one separate ward for HIV positive patients.

### 4.2. Participants

Participants who made up the target audience of the study were mainly medical staff specialized in infectious diseases, given their direct involvement in the treatment of the new disease, COVID-19. In order to have a better perspective on how telework influenced other doctors, we included other specializations within the hospital unit (psychology, ENT, gastroenterology, etc.) in the sample. The recruitment of the participants in the study was carried out using convenience sampling. 

In order to perform the investigation, a verbal consent from the hospital management was obtained. In the on-call report, the objectives of the research were presented to all the medical staff attending, with those willing to participate being scheduled for an interview. The interviews took place either at the hospital, in doctors’ offices, or by telephone. 

### 4.3. Data Collection

An interview guide (Appendix A) was developed by the research team. The interviews were conducted in February 2021, involving the participation of 20 medical staff. Each participant in the study was informed about the objectives of the study, how the interview would be conducted, the topics discussed, and the average time allocated for the interview. A consent form was signed by all participants in the study. Moreover, the study was conducted according to the ethical standards of the university. 

Interviews were carried out until the moment of thematic saturation [3], meaning until the information provided by them had been previously mentioned and no new topics were addressed. The average duration of an interview was 30 min. The interviews were transcribed and analyzed manually according to the objectives of the research. First the interviews were read, and then passages of interest were marked and labeled in categories [37].

The main categories that the data was categorized in were:

(a) Telework prior to COVID-19 pandemic
(b) The importance and adoption of telework during the pandemic
(c) Effects of telework on the workload of doctors
(d) Means of remote interaction with patients
(e) Limits of telework from a medical perspective
(f) Limits of telework from patients’ perspective
(g) Effectiveness of remote consultations
(h) Advantages and disadvantages of telework
(i) Influence of telework on the patient-doctor relationship

Research questions were explored by using an interpretative research approach, involving constant comparisons between interviewees’ answers [38]. The content analysis was applied in order to identify the specific themes, which were assessed in terms of frequency [39]. All members of the research team participated in the data interpretation. Online meetings were held in order to systematically and carefully report the content of each category and the overall findings, having a focus on the link between data collected and the reported results [40]. Moreover, all the authors gave input in the final analysis, ensuring the rigor and trustworthiness of the study. The results of the study are presented in the following section.

## 5. Results and Discussion

The sample consisted of 20 medical personnel (17 physicians and 3 psychologists), representing approximately 10% of the total hospital staff: 204 physicians and 7 psychologists at the time of the study. Regarding the gender distribution of the total number of medical personnel in the hospital, the ratio is 80% women and 20% men, the ratio being preserved in the investigated sample, i.e., 16 women and 4 men.

The 20 participants in the sample were structured according to the configuration presented in Table 1, 13 infectious disease primary physicians, 1 pediatrician primary physician, 1 endocrinologist primary physician, 1 gastroenterology primary physician, 1 otolaryngologist (ENT) primary physician, and 3 psychologists.

The research revealed that within the group of respondents consisting of physicians and psychologists, adding a telework component to their professional activity was generally seen as beneficial, but this change comes with both pros and cons.

One of the research questions was to understand how telework impacts the working schedule of doctors, and this is explained by the following results.

(a) The extent of using telework as a component of physicians’ schedules prior to the pandemic, its implementation situations, and specific context.

Telework used as part of the working schedule is a rather new element in doctor-patient relationships, which has developed in the context of the changes involved in the COVID-19 pandemic.

Respondents stated that before the outbreak of the pandemic, their working schedule consisted mostly of medical office consultations, while telework was used on a small scale, especially in the case of pregnant women, people with locomotive disabilities, or medical emergencies.

(b) The importance given to telework during the pandemic, and the time needed for doctors to reorganize their patient interactions.

According to the research results, prior to the epidemic, telework was used narrowly, in only about 10 percent of the daily schedule of medical staff. However, during the novel coronavirus pandemic, about 40 percent of the interactions between doctors and their patients were remote, even reaching as high as 60 percent among psychologists.

In the medical system, telework has been extended since March 2020, with respondents stating that they needed up to two months to adapt and organize these specific activities. It should also be noted that a number of healthcare professionals instantly adapted to telework requirements, and this helped to decrease SARS-CoV-2 infection rate among the healthcare staff of the hospital.

The health crisis induced by the COVID-19 pandemic was the trigger factor in adopting telework as a current method of dealing with patients’ needs and managing their chronic diseases, and the challenges of the current economic and social context have led to an immediate adaptation of the healthcare professionals to the particularities of a new reality in terms of patient interactions.

Despite the growing scope of telework and its importance in the daily activities of the medical staff, physicians and psychologists emphasize that they feel the need for a clear delimitation of telework tasks. Therefore, employers in the health system should establish, in a formal and regulated framework, which activities can be carried out via telework.

(c) Doctors’ perceptions regarding the effects of telework on total working time, and situations of professional overload.

Respondents mentioned several factors that have led to considerable changes in their medical activity and interaction with patients suffering from chronic diseases, since remote consultations were deployed amid the COVID-19 pandemic. 

A first factor, occurring as an effect of telework, is a major change in the effective working schedule of physicians and psychologists, as they have to work overtime and adapt to an extended working day. In the absence of a well-established consultation schedule, patients even contact healthcare professionals during evenings or on weekends: “*I have encouraged my patients to leave a message, and I try to answer them as soon as possible. I am not bothered by messages from patients on weekends or outside the working hours because I understand patients’ medical needs and the necessity of regular monitoring*” (MS).

Another factor that has led to the increase in the healthcare professionals’ working schedule is the large amount of time physically spent with patients that were diagnosed with COVID-19 and hospitalized in the red zone (infected zone). This is why doctors have to carry out part of their patient consultations via telework, sometimes outside the working schedule: “*extended physical working hours for COVID-19 patients means that most telework consultations, for patients with chronic diseases, have to be organized before or after the daily schedule, including on weekends*” (OS).

Consequently, telework proved to be very useful in managing patient relationships by allowing contacts and medical advice at a time when patients suffering from chronic diseases had difficulty accessing classic, face-to-face medical services. However, the lack of a formal framework to regulate the activity of healthcare professionals using telework also tended to generate a number of side effects. These effects were felt directly by the healthcare professionals in the form of overwork and additional job pressure, since teleworking activities are mainly carried out during their free time. In the long run, the lack of institutionally regulated telework policies and procedures can lead to dissatisfaction among both physicians and patients, who may receive insufficient care.

A possible solution to this situation may be the development of a patient relationship management system based on CRM technology to allow automation and optimization of appointments and remote consultations, while including them into the regular working schedule of physicians. Such a system should be adapted by CRM vendors or IT companies to the needs of the healthcare industry by designing specific functionalities to enable doctors’ access to patient medical history within specific databases, both inside and outside the medical unit, so as to allow telework from home. Access to patient profiles should further allow custom online communications and tailored medical solutions, according to the patient’s chronic diseases. Such an IT solution, containing data security and administration functions, requires a portal to be established in order to give doctors access to the CRM hospital system in a controlled and secure way. This should allow physicians and psychologists to administer their own users through the portal, to increase informational support and patient knowledge by directly documenting individual medical records, and, at the same time, ensuring the cybersecurity of patient information.

Another research question was finding out what methods are used by doctors in order to interact with their patients in remote consultations, as well as if these methods are generally applied or if they might not be appropriate in any situation and for any patient.

(d) Interaction methods used in remote consultations with patients and the technological applications involved.

Patient interactions in remote consultations take place mainly vocally (verbally), as well as by sending photos (especially in the case of pathologies with a dermatological component) or by using video, according to the patient’s need. 

There are also multiple communication technologies used in remote patient relationships. In this regard, respondents most often used WhatsApp and e-mail, without neglecting well-known platforms such as Google Meet, Zoom, or FaceTime: “*the patients choose the initial communication method when they first contact me, and then we agree on how to proceed. Patients usually start the conversation on WhatsApp or e-mail*” (RG). A possible solution in this case would be using a CRM software system to standardize these interactions in the physician-patient relationship, by using interactive communication methods that provide healthcare professionals with an optimal volume of information regarding patients’ health, which could be adapted according to various medical specialties. Choosing the communication channel, although convenient for patients, does not always allow for quality information inputs, which should be suitable for doctors to diagnose, monitor, or establish treatments relevant to the needs of their patients. This is particularly adequate for patients who prefer exclusive communication through verbal or written channels, and tend to avoid video interaction [41].

(e) Situations where telework consultations may not be relevant enough to accurately establish the proper therapeutic attitude or right patient diagnosis.

According to the research results, there are also situations when the physical attendance of patients at the medical unit is absolutely needed, especially in the case of medical emergencies. In most cases, the diagnosis and initial assessment for most diseases, as well as the physician’s assessment in case of a deterioration of the patient’s health condition, is performed in physical format, at the medical office: “*some patients may need a physical examination, an ultrasound or Fibroscan, emerging the need to physically attend the consultation*” (AS). Regarding the otorhinolaryngology specialty, consultations provided using telework cannot replace physical consultations: “*telephone consultations may bring a surplus of information, but they cannot cover the mandatory clinical examination*” (RD).

HIV-positive individuals might also need to physically visit the medical unit and have face-to-face communication with the doctor: “*especially HIV-positive women who gave birth and take care of a child ask many questions about their status or how to properly take care of the child, in addition to the information they were given when they presented themselves with the child at the hospital*” (MM), which is why they need additional information and regular meetings with the doctor. The HIV/AIDS phenomenon has deep psychological, social, and economic implications; an HIV-positive person wants to communicate with the doctor directly because there are certain intimate issues that they do not want to convey over the phone or through various applications.

(f) Physicians’ opinions regarding the categories of patients who encounter difficulties in participating in remote consultations.

Telework development in the healthcare sector is not without obstacles, given that some patient types might face real difficulties in participating in remote consultations. According to the research results, these patients are mainly elderly individuals, who usually do not have mobile phones with WhatsApp or other communication applications and cannot send or receive medical test results for specialized assessment: “*older people may have difficulties using technology, especially if they need to take pictures or send documents. However, they are usually helped by other family members*” (MM). Furthermore, “people with low education level have struggle communicating with the physician, especially regarding the installation and use of mobile phone apps” (SP, psychologist).

The fact that the research results identified various population categories who do not have access to modern technologies, perceiving difficulties in using online communication tools, or just facing different types of medical emergencies, leads us to consider that teleworking in the medical field cannot completely replace the physical, face-to-face doctor-patient interaction, neither nowadays, nor in the future, representing a complementary service that can be provided to patients, especially in the process of monitoring chronic diseases.

The final research question was to highlight the benefits and limitations of telework in medical services and in the doctor-patient relationship, as perceived by healthcare professionals. This is answered in the next research results.

(g) Doctors’ perception on the effectiveness of remote consultations compared to face-to-face consultations.

Results on the general perception of the effectiveness of telemedicine are unsurprising, with both physicians and psychologists considering face-to-face consultations more effective compared to remote patient interactions. According to respondents, in the case of telephone consultations, there is always a risk of losing medical information, as well as other important details about the patient health: “when the consultation takes place over the phone, there is a decrease in extremely important sources of information, such as facial expressions or gestures of the patients, and this leads to a longer time for acquiring all the medical information needed” (CC).

Additionally, respondents state that the time required for telework consultations is longer in most situations, especially when new patients are treated or new diagnoses are established: “*in many situations related to the initial diagnosis, it must be objectively confirmed through an in-person consultation*” (AA). At the same time, “*when there is a major difference between the patient’s perception of what is happening to him and the doctor’s perception of the medical information he receives from the patient, then the patient needs to have face-to-face interactions with the medical staff at the hospital, because his life may be endangered*” (MM).

(h) Advantages and disadvantages of telework during patient interactions, according to medical staff

The study was able to identify the ease with which the patient communicates with the physician as one of the major benefits of telework in doctor-patient relationships. This dialogue can be performed by just sending a text message, thus improving communication speed and decreasing waiting time for the patient: “*the patient benefits from quick and easy access to the consultation, without wasted time traveling to the hospital*” (VA).

Moreover, compared to traditional medical services, where, in some cases, scheduling a patient for a physical consultation at the medical office can take several weeks, the patient’s written message is directly sent to the physician, and the patient’s request is usually resolved on the same day.

Adding to the research results of the impact of the COVID-19 pandemic on the population in terms of reducing travel, physical interactions, and mobility, the benefits of teleworking and telemedicine for patients can be understood in a broader context, primarily from the perspective of ensuring the continuity of medical services and the patients’ access to physicians’ expertise. The patient’s anxiety can be decreased by practicing telework, given that communication with the physician takes place from home. This diminishes social exposure and the psychological costs for individuals, and allows them to save time and travel costs, which are specific to physical, face-to-face interactions.

According to the research results, the limits of telework mainly affect the medical staff’s wellbeing. One significant disadvantage is related to overloading doctors’ schedules, who have to carry out remote consultations even outside of working hours and on weekends. Teleworking leads to a higher daily workload because the medical staff, in addition to the intensive physical consultations carried out with the COVID-19 patients, must carry out remote consultations with the chronically ill, non-COVID-19 patients. All this additional effort can cause a lower yield in medical performance, which makes it necessary to better plan telework activities and properly integrate them with the physical consultation program of physicians.

Patients suffering from chronic diseases, such as hepatitis, HIV/AIDS, or diabetes might also experience specific barriers in telework consultations; some examples are: “possible inaccurate diagnosis given the complex medical history, difficulty in understanding the treatment program, difficult anamnesis situations” (MS). At the same time, in the case of psychological consultations, “*the main disadvantage is related to the human factor—difficulties in monitoring the behavioral reactions of patients, limits in performing physiological assessments by pencil-paper tests or non-verbal behavior*” (CC).

Consequently, the difficulties faced by physicians in observing the details of patients’ health and their non-verbal behavior, the poor cooperation of certain patients, or misunderstanding of the doctor’s recommended treatment are important limitations of teleworking in the medical services sector, which raise questions about the widespread adoption of these practices in the future. The main argument in this regard is the risk of misdiagnosis and inefficient treatment, with possible negative effects on patients’ health.

(i) Physicians’ perception of how telework influences patient relationships, and how they see the future of telework in the medical field after the pandemic.

Overall, doctors and psychologists perceive the results of remote consultations and the extent to which they managed to meet patients’ health requests during the pandemic rather favorably, but, at the same time, they identify the need to optimize the appointment process to avoid an overload of the long-term working schedule.

The healthcare professionals are aware that teleworking was necessary in the case of patients suffering from chronic diseases who needed ongoing treatments. Moreover, the feedback of these patients was generally positive, given the context of adopting telework. Of course, there are also some weaknesses that can cause potential dissatisfaction, such as “*the impossibility of remotely carrying out the complete medical assessment, including analysis and/or imaging evaluation via telemedicine, which is why a first telework consultation is performed, followed by the recommendation for the patient to carry out additional physical assessments, and after that another telework consultation is performed in order to interpret the result of the investigations*” (OS).

The advantage of an adequate level of patient satisfaction regarding the way remote consultations are carried out, on the one hand, and the limitations of providing complete medical services, on the other hand, make it necessary to continue using telemedicine, but only as an additional method to face-to-face, physical, medical consultations and investigations.

On a social level, respondents believed that telemedicine caused an increase in people’s availability to use medical services, but there is also a part of the population that prefers face-to-face health services and who seem to be late adopters of online interaction methods. According to the opinion of some psychologists, “*teleworking has led to an increase in patient availability to use psychological services, precisely because of the ease of contacting a specialist in the field, and also due to lower time and money constraints*” (RP).

Therefore, the advantages that telework and telemedicine bring in terms of accessibility and availability of medical services for patients are high enough, and, if these working methods are formally adopted and properly regulated by medical institutions, it is expected to also bring economic and productivity benefits.

Our research results also prove the intention of different healthcare professionals to continue performing telework consultations after the end of the pandemic. According to the opinion of the majority of respondents, implementing a hybrid system would be beneficial, based on both face-to-face consultations at the medical unit and remote interactions, depending on the patient’s profile and healthcare needs. The latter may primarily target patients with stable chronic diseases. In such a system, it is recommended that the initial diagnosis and assessment be made in person at the hospital, followed by remote monitoring via telemedicine, except where re-assessment is required.

## 6. Conclusions

The COVID-19 pandemic has led to many social and economic changes. Thus, in order to avoid the spread of the virus, many states have taken measures to limit people’s interactions, partially or totally restricting educational, cultural, or recreational activities. This epidemiological background has also impacted the labor market, which had to re-assess its processes, discriminate between essential and non-essential staff, and decide if teleworking methods are safer for employees and employers. Telework became widely accepted due to the intense development of ICT, which has enabled the transition from office work to home work under good conditions.

Our paper discusses an area where face-to-face interaction has traditionally been the essence of how healthcare services are provided. As in the case of the education system, the health system had to adapt along the way to the new context and integrate telework in different proportions. If before the pandemic, telemedicine was a tool used only to address the rural population or from hard-to-reach areas [42], now it has become a real way of treating chronic patients.

Our study highlighted the impact that telework had among the medical staff in Romania and underlined the importance of developing a formal mechanism for carrying out this type of activity, in the current pandemic context. The information obtained through qualitative research is valuable in terms of revealing the ability of the medical system, and medical staff, to adapt to changes in the external environment in order to ensure the sustainability of the health service for patients suffering from chronic diseases.

The qualitative research conducted among the medical staff of a state-owned hospital, directly involved in the treatment of COVID-19 patients, showed how the medical staff adopted telework, as well as the advantages and disadvantages that came with this solution. We can also see the diversity of communication methods used by the medical team in patient relationships. If in countries such as Pakistan, only one way of remote communication technology was used in patient relationships—the WhatsApp application [43]—in Romania we noticed a high degree of flexibility in choosing the interaction platform, depending on the patient’s level of technological literacy.

This paper extends the level of knowledge regarding the process of telework adoption within the medical system. It proves how the COVID-19 pandemic and the development of ICT tools have been the trigger factors in speeding up this process. The paper also provides a better understanding of how physician-patient interactions work in order to design a specific procedure dedicated to telework in this area, considering its advantages and disadvantages. At the same time, the research highlighted a phenomenon of overburdening of medical staff during the pandemic, given their multiple activities, involving both healthcare services provided to COVID-19 patients within the hospital, and remote consultations or online interactions with patients suffering from chronic diseases. Moreover, we note that telework activities were not usually included into the daily schedule of the medical professionals, but rather outside of their working hours. For this reason, situations of overload, demotivation of the medical staff, or burnout may occur.

In order to decrease the negative consequences, as well as to strengthen the benefits of telework within the Romanian medical system, specific actions are recommended based on the research results. A first action initiative should come from the macro, governmental level, where additional steps are needed to entirely clarify the formal framework for telework implementation, according to both the medical specialization and the physical working schedule of doctors, so as to avoid overburdening situations for the medical staff or insufficient care for some patients. A second call for action is needed at the micro level, within hospitals or other types of medical units, where managers need to understand the need to implement a patient relationship management system based on CRM technology. We consider that a CRM system adapted to the internal processes of medical units would allow the secure access of doctors to patient profiles, in a controlled and timely manner, in order to enable tailored medical treatments and customized remote communications, while also optimizing the appointment process. In the long run, CRM technology could help in better predicting the working schedule of each doctor, avoiding overload issues by better planning telework activities and properly integrating them with the physical consultation program of physicians. Last but not least, the automation of medical activities that can be carried out through telework might lead to economic and productivity benefits for the medical unit.

Limits of the research are determined by the characteristics of qualitative studies, where the exploratory nature is consistent, not considering the representativeness of the research for the entire medical system. We consider it useful to extend this research approach to include other types of hospitals with different degrees of involvement in the treatment of COVID-19 patients, as well as at the level of general practitioners’ offices.

## Figures and Tables

**Table 1:** The structure of the researched group.

No.	Participant Code	Sex	Seniority in Work	Specialization
1	MM	F	>30 years	infectious diseases primary physician
2	VA	F	>25 years	infectious diseases primary physician
3	AA	M	>25 years	infectious diseases primary physician
4	AS	F	>20 years	infectious diseases primary physician
5	OM	M	>20 years	infectious diseases primary physician
6	MS	F	>15 years	infectious diseases primary physician
7	DM	F	>15 years	infectious diseases primary physician
8	MO	F	>15 years	infectious diseases primary physician
9	OS	F	>10 years	infectious diseases primary physician
10	AD	F	>10 years	infectious diseases primary physician
11	DA	M	>10 years	infectious diseases primary physician
12	CS	F	>10 years	infectious diseases primary physician
13	CI	F	>10 years	infectious diseases primary physician
14	DA	F	>25 years	pediatrician primary physician
15	CO	F	>25 years	gastroenterology primary physician
16	RG	F	>15 years	endocrinologist primary physician
17	RD	F	>10 years	otolaryngologist (ENT) primary physician
18	SP	M	>25 years	psychologist
19	RP	F	>25 years	psychologist
20	CC	F	>20 years	psychologist

Source: research data analysis.

## Data Availability

Not applicable.

## Appendix A. Interview Guide

**Q1:** Before the outbreak of the COVID-19 pandemic, did your working schedule include telework? If yes, in what situations and in what proportion of your patient working time?
**Q2:** During the coronavirus pandemic, how many patient interactions have been teleworked? How much time did you need to organize your remote consultations? Is telework regulated by your employer?
**Q3:** Does telework affects your normal working schedule? Please describe the changes in your working schedule compared to the period before the pandemic.
**Q4.** How do telework consultations take place? What applications or technologies do you use to communicate with your patients? What interaction methods are preferred by your patients?
**Q5:** Describe the situations when patient physical attendance at the medical unit is needed and when remote consultations are not relevant or possible.
**Q6:** Which categories of patients experience difficulties in participating in remote consultations and what are their issues about?
**Q7:** How effective do you think remote consultations are compared to face-to-face consultations, in terms of time and dialogue with the patient?
**Q8:** What are the advantages and disadvantages of telework in your medical activity? What about the benefits and limitation for patients?
**Q9:** What are the implications of telework in the doctor-patient relationship? Do you think you have been able to meet the needs of your patients through remote consultations and what is their feedback?
**Q10:** On a social level, do you consider that telework and telemedicine causes an increase or decrease in the availability of the population to use medical services?
**Q11:** How do you see the perspectives of telework in the medical field after the COVID-19 pandemic?
